# Hydrophobia Treated with Chloroform

**Published:** 1849-01

**Authors:** 


*Hydrophobia treated with Chloroform.*—A case of hydrophobia, treated unsuccessfully with Chloroform, is reported in the American Journal of the Medical Sciences, by Dr. Hartshorne.

The boy was thirteen years old. Was taken sick about six weeks after being bitten by a dog proved to behave as if rabid. Had high fever, which had no remission, and yet no characteristic of typhus or typhoid fever; he was not deaf; had no tympanitis; no petechia; but had throughout, excessive sensibility to impressions, particulary those of cold, and about the face; had a difficulty, at times incapability, of swallowing, caused by a spasm which made a violent respiration simultaneous with the attempt to swallow, and thus impeded the latter and endangered the passage of liquid into the windpipe; which symptom was constant, and belongs to no ordinary type of disease; had injections of laudanum, ℨss or ℨj, repeated again and again with no effect: and breathed chloroform for hours, with only short intermissions, with no more effect than to tranquilize him, and produce natural sleep, from which he was, once at least, roused by a return of wild delirium: and yet had no sign whatever of tetanus: no possibility of mania-a-potù: and very little and only occasional complaint of pain: which pain, when it occurred, except in the first stage, was in the arm which had been bitten by the dog; had also salivation, constant watering of the eyes, and finally vomiting: and whose delirium was, until quelled, of a malicious and furious character, or one of dread, horror, and distress: ending fatally at the end of the seventh day.

What name should be given to such a case? "Hydrophobia" is not appropriate, because in this and other cases they *desire* drink, but are incapable of benefitting by it: would not *dyspotia*, or rabies dyspotia be more correct?

It is a higly important fact, that the inhalation of chloroform, persevered in at intervals, did master the violent delirium and horror of the case. Dr. Smiley, of Philadelphia, and Dr. Stone of Easton, have already noticed the same effect; although their cases also were fatal. This removes, at least one element of evil from the disease. And if any remedial agency can ever give hope of *cure*, it mus be, when aided by other means, one which has such control over the symptoms, attained hitherto by no other medicine.

We were deterred from depletion by the lancet by the supposed analogy to tetanus, in which stimulation is mostly requisite and useful. But free bleeding is said to have effected one or two recoveries; and has *a priori* plausibility in its favor, as the disease is inflammatory both in symptoms and postmortem appearances. We were unwilling to use counter irritation to the spine, from the idea that death was certain, that those means only were, therefore, justifiable, which lessened or did not increase suffering. And the early resort to chloroform was prevented by respect for the doubts of those who at first were inclined to regard it as a case of fever.

With a distinct diagnosis, should another case present, in recollection of the mitigation already obtained—would not the practitioner be justified in commencing with venesection; using cloroform, with discretion, from an early period; and applying some powerful revulsive, aided by a narcotic, to the nucha? Dr. R. S. Wodrop proposed vesication, followed by the free application of a salt of morphia. Perhaps *acupuncturation with aconitia*

might prove more powerful. This treatment has been found successful in obstinate neuralgia. I used a sort of cataplasm of tinct. rad. aconit. to the above case. An excessive sensitiveness to incident impressions, and consequently excessive motory action of particular muscles, *including the diaphragm* obviously in the case, as well as those of the throat, seems to present. Remedies which lessen sensation are the ones, then, which may mitigate the disorder at least. In France, the vapor bath is asserted to have wrought a cure; its efficacy should be tried, and might be aided, as was contemplated in our case, by medication with such articles as tobacco, chloroform, aconite, to produce a powerfully antispasmodic fumigation of the whole surface of the body. Prof. S. Jackson advised bathing in warm oil. When swallowing is impossible, the stomach tube might be inserted through the nostril, as done by Dr. Joseph Hartshorne, a number of years since, in the hospital; and life also supported by nutritious enemata.

But careful reflection will only deeply impress the conclusion, that with all the means that man can devise, the malady is one which affords less hope of cure, decidedly, than phthisis; and that prevention by immediate excision (or cauterization?) of the bitten part, is the only safeguard against its result.—*Western Jour. of Med.*

